# Relaxation Estimation of RMSD in Molecular Dynamics Immunosimulations

**DOI:** 10.1155/2012/173521

**Published:** 2012-09-16

**Authors:** Wolfgang Schreiner, Rudolf Karch, Bernhard Knapp, Nevena Ilieva

**Affiliations:** ^1^Section of Biosimulation and Bioinformatics, Center for Medical Statistics, Informatics, and Intelligent Systems (CeMSIIS), Medical University of Vienna, Spitalgasse 23, 1090 Vienna, Austria; ^2^Institute for Nuclear Research and Nuclear Energy (INRNE), Bulgarian Academy of Sciences, 72, Tzarigradsko Chaussee, 1784 Sofia, Bulgaria

## Abstract

Molecular dynamics simulations have to be sufficiently long to draw reliable conclusions. However, no method exists to prove that a simulation has converged. We suggest the method of “lagged RMSD-analysis” as a tool to judge if an MD simulation has not yet run long enough. The analysis is based on RMSD values between pairs of configurations separated by variable time intervals Δ*t*. Unless RMSD(Δ*t*) has reached a stationary shape, the simulation has not yet converged.

## 1. Introduction

Mathematical modeling and computational biology have proved extremely successful in the efforts to understand the mechanisms of immunologic reactions, for example, in modeling the T-cell proliferation dynamics following hepatitisC, HIV infection [1], or the growth of an immunogenic tumor [2]. Methods of statistical mechanics may successfully be applied in order to quantitatively understand the immune system [3, 4], and different modeling approaches are adequate for acquired immunity, as surveyed in a seminal paper by Perelson and Weisbush [5]. Modeling and simulation can be performed on different levels, starting at the top level with agent-based models [6, 7] for the cooperation of numerous large biomolecules in the formation of the immune synapse [8–11] down to more detailed models of antigen binding [12], recognition, and signaling [13–20]. T-cell proliferation modulated by interleukin2 has been simulated [21]. Applications aiming to predict the reaction to epitopes [22] improved vaccine design [23], vaccination against tumors [24], and for improved patient care have been devised [25–27], also in personalized medicine [28]. In particular, the mechanisms how T-cell receptors (TCRs) detect antigen peptides(p) presented by major histocompatibility complex (MHC) molecules can be investigated by molecular dynamics(MD) on a molecular or even atomic level [29–32]. However, TCRpMHC complexes are huge protein complexes, which have to be studied in water, which adds an even larger number of atoms to model and simulate. For these reasons, sufficiently long simulations are mandatory in order to obtain realistic results. This is even more so since molecular recognition phenomena may operate on rather long timescales as compared to the length of usual MD trajectories. Hence, efforts to assess sampling quality are mandatory in such simulations.

Given the trajectory of an actual MD simulation, it is clear from first principles that no formal check whatsoever can prove that complete sampling has been achieved. If parts of the phase space have not been visited yet, there is no possibility that this becomes evident from looking only at data from those other parts that have been visited. Zhang et al. [33] proposed a formal approach to decompose the phase space into Voronoi polyhedra [34, 35].

At any rate, formal checks of sampling quality can only draw on simulation data already produced and *at the most detect possible shortcomings* of those trajectories. The situation is analogous to tests for the randomness of pseudorandom-number generators [36]. One can never prove randomness as such. It is only possible to detect specific deviations from randomness, such as serial correlations—and any such detection works only with a preselected error rate.

Likewise, MD trajectories may be investigated for specific markers of nonrandomness, the most important type being trends of energy and molecular deformations. This work focuses on the latter, deviations in shape being quantified via the *root mean square deviation* (RMSD) [37] at time *t*_2_ with respect to a given reference structure at time *t*_1_:

(1) $$\begin{array}{c} {\text{RMSD}\left(t_{1},t_{2}\right)=\left[\frac{1}{N}\sum_{i=1}^{N}{||x_{i}\left(t_{2}\right)-x_{i}\left(t_{1}\right)||}^{2}\right]}^{1/2}, \end{array}$$

where **x**_*i*_(*t*) is the position of atom *i* at time *t* and *N* is the total number of atoms in the molecule.

**Figure 1:**
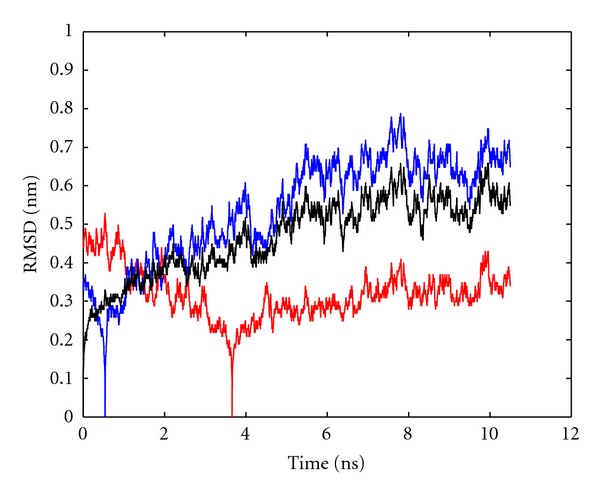
RMSD as a function of time for trajectory1 (10 ns). Black curve: RMSD between first configuration and all successive ones. Blue curve: for a given configuration (*t*_*j*_), RMSD to each of the other configurations of the same trajectory was computed and averaged, yielding $\overset{-}{\text{RMSD}}(t_{j})$. That value of *t*_*j*_ for which $\overset{-}{\text{RMSD}}(t_{j})$ is maximum is then adopted as a reference (*t*_1_) to plot RMSD values of the whole trajectory. Red curve: as blue curve, but for minimum $\overset{-}{\text{RMSD}}(t_{j})$. Note that the RMSD between the reference configuration and itself is zero by definition.

Often, the first frame of a trajectory (*t*_1_) is used as a reference, and values of RMSD(*t*_1_, *t*_2_) are computed for all successive (*t*_2_ > *t*_1_) frames; see the black curve in Figure 1. RMSD monitored this way shows large rapid fluctuations on top of long-term variations and jumps. Generally, it is difficult to identify such long-term variations, which may relate to functional modes. A recent study [38] reflected the insufficiency of visual RMSD inspection alone.

Extensive work has been published on using the RMSD for the characterization of structural changes, drifts, and trends; see [33] and the references cited therein. Grossfield and Zuckerman [39] gives a seminal conceptual discussion of ergodicity, absolute and relative convergence and proposes checks for overall sampling quality. Block averaging was proposed [40] to reduce short-term fluctuations and to obtain more reliable indicators of long-term trends. However, averaging RMSD over all configurations within a block involves many different time intervals Δ*t* = *t*_*j*_ − *t*_*i*_, thus reducing the specificity of the resulting average for one particular time interval.

For this reason we consider the RMSD between pairs of configurations separated by a constant time lag Δ*t* as described in Section 2.2.1. In this way we obtain more stable estimates (averages) which are nevertheless perfectly specific for a given time interval Δ*t*.

## 2. Materials and Methods

### 2.1. Molecular Dynamics Simulations

#### 2.1.1. Employed Structures

We applied the proposed methodology to a total of 6 MD simulations. For this purpose we used 2 different TCRpMHC complexes: an immunogenic wild-type peptide bound between TCR/MHC and the same TCR/MHC with a less immunogenic mutant peptide. We employed the crystal structure of the LC13 TCR bound to HLA-B*08 : 01 and the Epstein Barr Virus peptide FLRGRAYGL. This structure is available from the Protein Data Bank (PDB) [41] via PDB accession code 1mi5 [42] and is referred to as wild-type. For the mutant complex we substituted the side chain of tyrosine at position 7 of the peptide to alanine (Y7A). This was performed using SCWRL [43] since we could previously show that this tool is most appropriate for mutations in pMHC complexes [44, 45].

**Figure 2:**
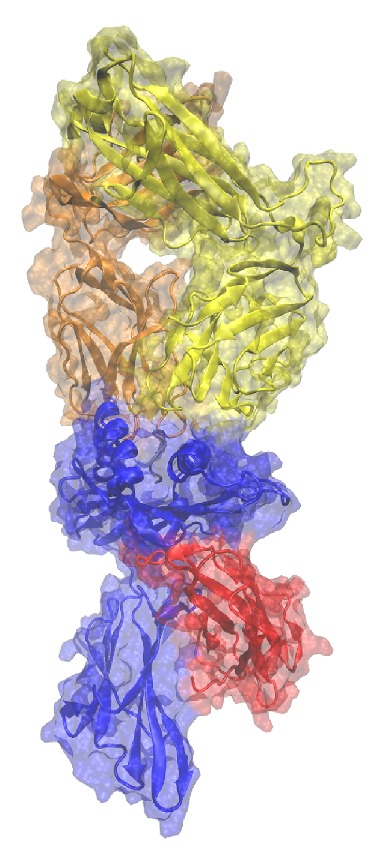
Illustration of the LC13 TCR in complex with HLA-B*08 : 01. Blue: MHC, red: *β*2-microglobulin, gray: peptide, orange: TCR alpha-chain, yellow: TCR beta-chain.

The LC13 TCR in complex with the FLRGRAYGL peptide and HLA-B*08 : 01 is an ideal test set for molecular dynamics simulations since this complex was crystallized and described in its parts and as a whole. Initially Kjer-Nielsenetal. crystallized the TCR [46] and the MHC [47] separately while they published a structure of the whole TCRpMHC system [42] afterwards. These available data give substantial insight into the LC13/EBV/HLA-B*08 : 01 system and led to the choice of our wild-type and mutated system for this study. The whole system is illustrated in Figure 2.

#### 2.1.2. Molecular Dynamics Simulation Protocol

The wild-type and the mutant complex were simulated in independent runs for 10, 50, and 200 ns yielding a total of 6 simulations (see Table 1). The following protocol for the simulations was employed. First, we minimized the energy of the systems using a steepest descent method. Then we immersed the complexes in explicit SPC [48] artificial water baths allowing for a minimal distance of 2 nm between the box boundary and the protein. Next, we warmed the complex up to 310 K using position restraints. Finally, we carried out the simulations using GROMACS4 [49] and the GROMOS96 force field [50]. All further parameters were set in accordance with [51].

#### 2.1.3. RMSD Calculation

After the simulations were finished, we calculated the RMSD values for each configuration in a given trajectory with respect to every other configuration of the same trajectory using the standard *g_rms* function of GROMACS. This yields an *n* × *n* matrix of RMSD values where *n* is the number of configurations in the trajectory.

### 2.2. RMSD between Configurations of Trajectories

#### 2.2.1. Averaging and Modeling of Lagged RMSD

Given one configuration **x**(*t*_*i*_) of an MD simulation as a reference, the RMSD to some other configuration **x**(*t*_*j*_) may be considered a “distance measure” along the time interval Δ*t* = *t*_*j*_ − *t*_*i*_. If Δt is short enough, it may be shifted along the whole trajectory and RMSD values be sampled. The average $\overset{-}{\text{RMSD}}\left(\Delta t\right)$ is characteristic for the difference between configurations separated by Δ*t* in the particular simulation run considered.

Small values of Δ*t* characterize configurations close to each other in time. Increasing Δ*t* means to compare configurations more distant to each other in time, which are—intuitively speaking—“less related” to each other. This fact should be reflected in $\overset{-}{\text{RMSD}}(\Delta t)$ for increasing values of Δ*t*.

As Δ*t* increases, dependences should diminish and approach the level of “unrelated” or “independent” configurations. In order to quantify such a saturation trend, we applied the *Hill equation* [52]:

(2) RMSD−(Δt)=a·Δtγty+Δtγ,

where the parameter *a* reflects the maximum value to which the function is asymptotic (“plateau value” $\overset{-}{\text{RMSD}}\left(\Delta t\to \infty \right)$), *τ* is the time lag Δ*t* for which $\overset{-}{\text{RMSD}}\left(\Delta t\right)=a/2$ (i.e., the value of Δ*t* for which half-saturation is achieved), and the *Hill coefficient γ* is a parameter that determines the shape, that is, the level of sigmoidicity, of the model functions. The parameters were estimated by fitting the *Hill equation* (2) to the measured values of $\overset{-}{\text{RMSD}}\left(\Delta t\right)$ using the *nlinfit* function as implemented in the *Statistics Toolbox* of MATLAB(Mathworks, Natick, MA, USA). The maximum time lag Δ*t* was chosen half the total simulation time of the respective trajectory.

#### 2.2.2. Assessing the Influence of Initial Conditions

The initial phase *t* ≤ *t*_offset_ of each MD simulation strongly depends on the starting configuration, usually a crystal structure, which can by no means be representative for a configuration obtained from a trajectory. This is even true after energy minimization and warming up. Hence, if the initial phase of an MD trajectory is included in $\overset{-}{\text{RMSD}}\left(\Delta t\right)$, a bias will result. This is not only true for individual values of $\overset{-}{\text{RMSD}}\left(\Delta t\right)$ but also for the parameters estimated from the fit. In particular, the limiting plateau value $a=\overset{-}{\text{RMSD}}\left(\Delta t=\infty \right)$ will also be biased and depend on *t*_offset_: in fact, *a* = *a*(*t*_offset_). We modeled this dependence as

(3) $$\begin{array}{c} a\left(t_{\text{offset}}\right)=a_{0}+\beta \cdot \exp\left(-\lambda \cdot t_{\text{offset}}\right), \end{array}$$

where *a*_0_ is the limiting value, *a*_0_ = *a*(*t*_offset_ = *∞*), and *β* and *λ* are scaling parameters. Note that *a*_0_ is an extrapolated estimate for $\overset{-}{\text{RMSD}}\left(\Delta t=\infty \right)$ and *t*_offset_ = *∞*, that is, the estimate for an RMSD between two totally unrelated configurations of a trajectory, independent of initial phase effects.

Values for *t*_offset_ were selected in the interval 0 ≤ *t*_offset_ ≤ *t*_max⁡_/2, where *t*_max⁡_ denotes the total simulation time of the respective trajectory.

## 3. Results and Discussion

### 3.1. Convergence of RMSD with Increasing Time Lag

**Figure 3:**
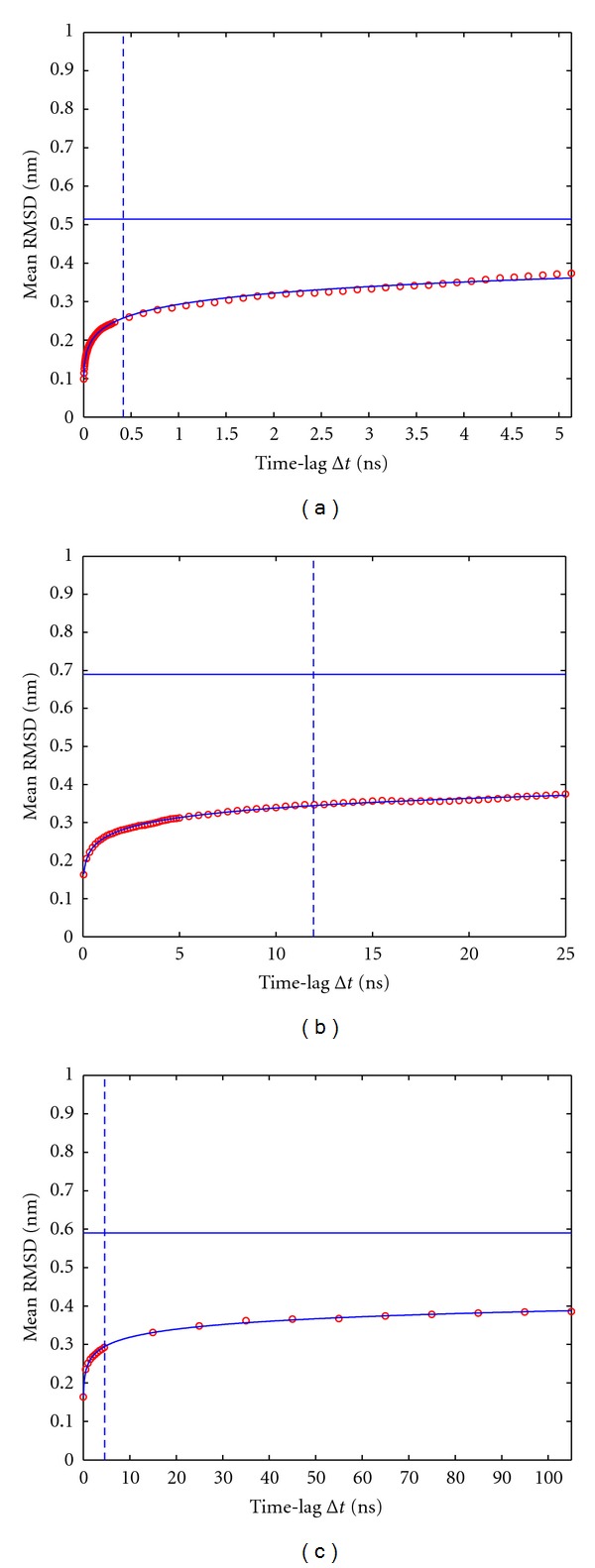
Dependence of mean RMSD on the time lag Δ*t* for *t*_offset_ = 0. (a) 10ns trajectory1, (b) 50 ns trajectory2, (c) 200 ns trajectory3; see Table 1. Note that due to the different total durations of the simulations, the maximum time lag considered (equal to half the simulation length) varies. Vertical lines indicate the time interval *τ*, for which half-saturation is achieved; see also (2). The horizontal line indicates the estimated plateau of the mean RMSD, corresponding to the parameter *a* in (2).

Each panel of Figure 3 shows a representative plot (for reasons of conciseness we display results in the figures only for trajectory 1–3) of mean RMSD values (red circles, *y*-axis) obtained between configurations of one MD trajectory (see figure caption and Table 1), separated by respective time lags Δ*t* (*x*-axis). As the time-lag between configurations increases, mean RMSD approaches a plateau.

Fits of the model (2) to the values of $\overset{-}{\text{RMSD}}\left(\Delta t\right)$ are displayed as solid lines. Parameters obtained from the fits can readily be interpreted as follows. The estimate for parameter *a* in (2) represents the limiting value of $\overset{-}{\text{RMSD}}\left(\Delta t\right)$ and is indicated by the horizontal line. The estimate of the parameter*τ* corresponds to a “characteristic” (“half-saturation”) time interval Δ*t* and is shown as a vertical line in the plots.

Note that the initial phase of each trajectory strongly determines the shape of $\overset{-}{\text{RMSD}}\left(\Delta t\right)$ which should be properly represented by the fitted model. In contrast, the remainder of each trajectory is characteristic for the long-term trend of $\overset{-}{\text{RMSD}}\left(\Delta t\right)$. Although it shows much smaller changes in RMSD, the remainder contains naturally by far more data points as compared to the initial phase. To achieve an appropriate balance, we increased the lag length in small steps during an initial phase (cf. the initially dense succession of red circles in Figure 3) and in larger steps (more loose succession of circles) later on. This procedure puts increased weight on the data points for the initial phase.

### 3.2. Influence of the Offset

**Figure 4:**
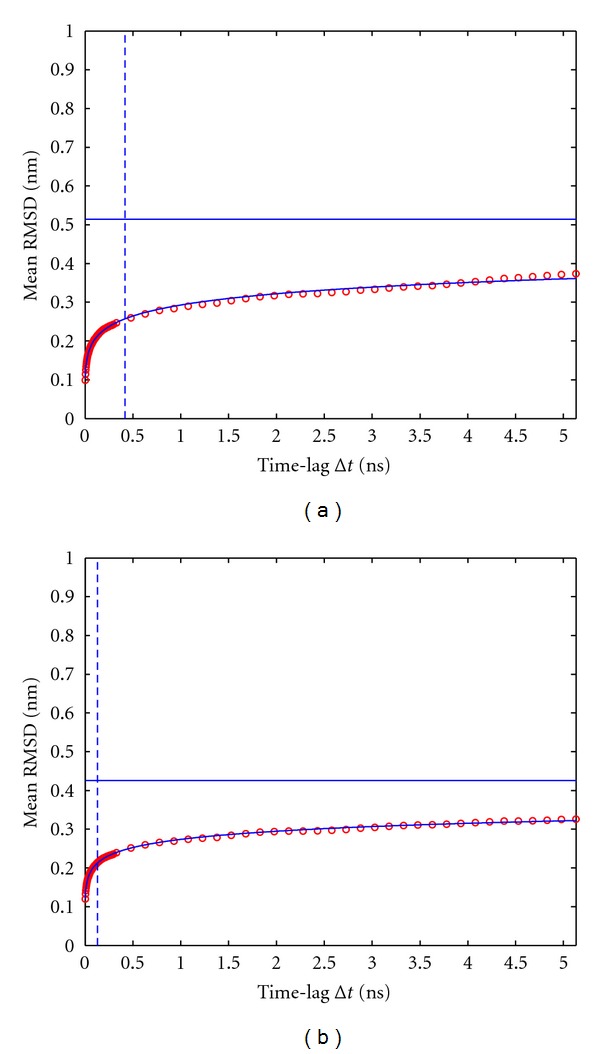
Dependence of mean RMSD on *t*_offset_ for trajectory1 (10 ns). (a): *t*_offset_ = 0 ps, (b): *t*_offset_ = 200 ps. Vertical lines indicate the time lag *τ*, for which half-saturation is achieved; see also (2). The horizontal line indicates the estimated plateau of the mean RMSD, corresponding to the parameter *a* in (2).

Applying different temporal offsets *t*_offset_ before starting to analyze the respective trajectories changes the dependence of $\overset{-}{\text{RMSD}}\left(\Delta t\right)$ as a function of the time lag Δ*t*; see the typical results displayed in Figure 4.

**Figure 5:**
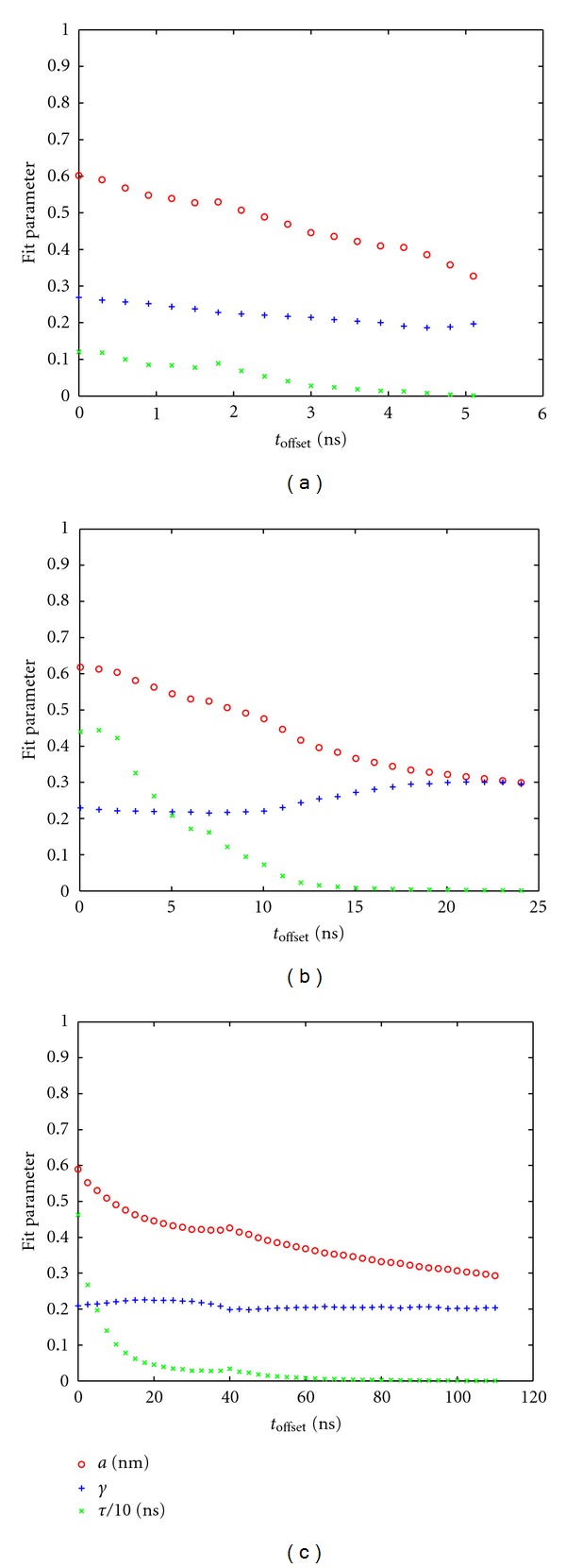
Increasing *t*_offset_ changes fitted parameters of $\overset{-}{\text{RMSD}}(\Delta t)$, (2). (a) 10 ns trajectory, (b) 50 ns trajectory, (c) 200 ns trajectory; see Table 1.

The larger the offset *t*_offset_ from the start configuration, the smaller the time lag Δ*t* necessary for the system to level off to its $\overset{-}{\text{RMSD}}$ plateau. The exemplary display of the dependence of fitted parameters on *t*_offset_, as shown previously, is systematically analyzed as follows; see Figure 5. With increasing *t*_offset_ both the plateau value *a* and the half-saturation parameter *t* decrease, whereas the shape parameter *γ* is fairly constant and almost independent of *t*_offset_.

**Figure 6:**
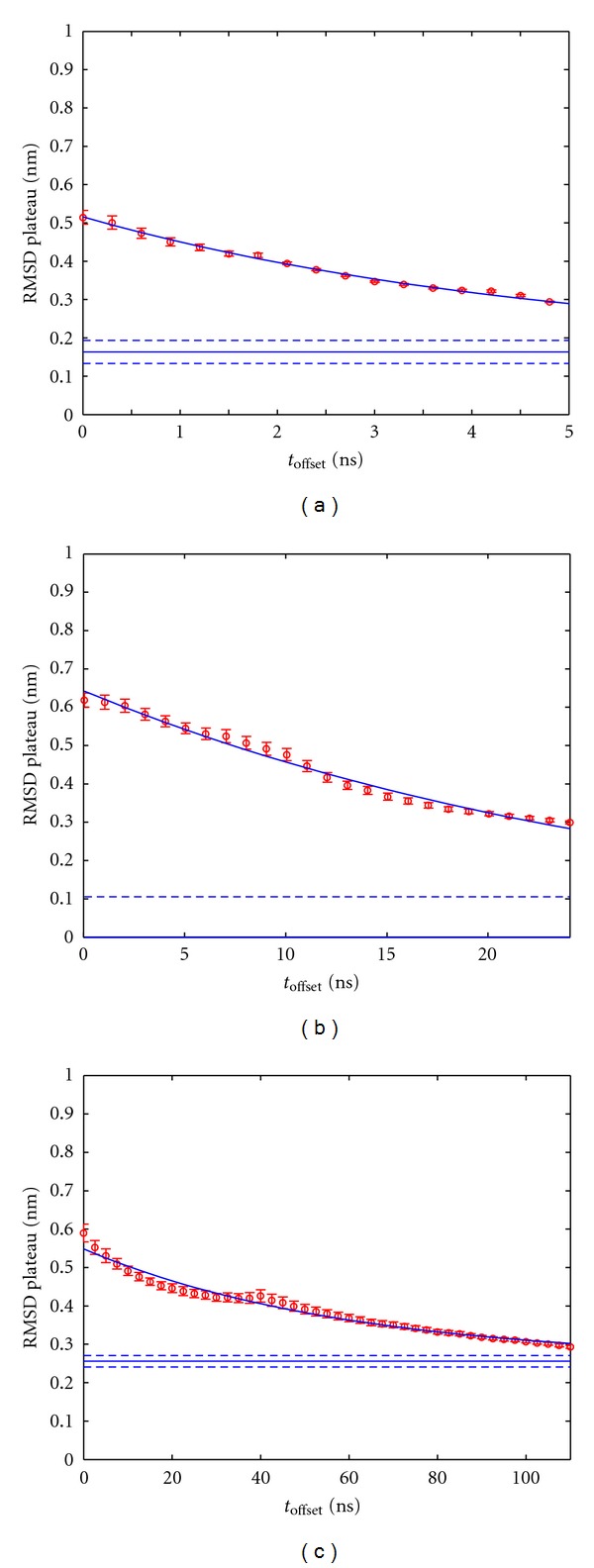
Fit and extrapolation of $\overset{-}{\text{RMSD}}$ plateau values for increasing *t*_offset_. (a) 10 ns trajectory1, (b) 50 ns trajectory2, (c) 200 ns trajectory3; see Table 1. Red circles represent plateau values *a*(*t*_offset_) the error bars denote their asymptotic standard errors. The solid blue curve shows a nonlinear least-squares fit of (3) to the plateau values *a*(*t*_offset_). From the latter fit the limiting plateau value *a*_0_ = *a*(*t*_offset_ = *∞*) was extracted and is shown as a solid horizontal line together with its asymptotic standard error as obtained from the fit of (3).

In a next step the systematic dependence of the extrapolated plateau on *t*_offset_ was fitted via (3); see Figure 6. The monoexponential decay model of (3) represents the most parsimonious choice, given the shape of the simulation results as displayed in Figure 6.

Figures 6(a)–6(c) illustrates the influence of the offset *t*_offset_ from the start configuration on the respective $\overset{-}{\text{RMSD}}$ plateau values as estimated from the parameter *a* of the model (2).$\;\overset{-}{\text{RMSD}}$ plateau values tend to decrease with increasing *t*_offset_.

## 4. Conclusions

For molecular dynamics simulations of proteins, questions of “convergence” and sampling are important issues if sensible conclusions are to be drawn from such simulations. Since convergence of a particular simulation (in the sense that statistical sampling of the phase space is complete enough with respect to the specific phenomena studied) cannot be judged in advance [39], various techniques have been advised to demonstrate that a simulation has not yet converged [37, 53–56].

Here, we propose a method to assess if a simulation has *not* yet run long enough, based on RMSD analysis of successive configurations of a given MD trajectory, separated by time lags Δ*t* of varying length (up to half the total simulation time). As long as a simulation has not yet converged, the shape of the function $\overset{-}{\text{RMSD}}\left(\Delta t\right)$ still considerably depends on *t*_offset_, that is, the time point along the trajectory, where the analysis interval Δ*t* starts. As *t*_offset_ is large enough, the shape of $\overset{-}{\text{RMSD}}\left(\Delta t\right)$ becomes stationary; that is, in Figure 4 the shape of the mean RMSD curve is different in the left and right panel, but further increasing *t*_offset_ would not further change its overall appearance. This is also reflected in the respective model parameters, converging to constant values for large values of *t*_offset_; see Figure 5.

To describe this Δ*t* dependence of the mean RMSD values,$\;\overset{-}{\text{RMSD}}\left(\Delta t\right)$, for a given *t*_offset_, the Hill function was used. This function type is frequently applied in enzyme kinetics to model saturation phenomena together with the number of reactive sites on an enzyme. In the present work, the Hill function proved flexible enough to model the functional form of $\overset{-}{\text{RMSD}}\left(\Delta t\right)$ and to identify the respective parameters (plateau value, shape parameter, and half-saturation time). Contrary to a simple exponential saturation function, the Hill function is able to model different levels of sigmoidicity.

In order to quantify the influence of the initial conditions and the equilibration phase on $\overset{-}{\text{RMSD}}\left(\Delta t\right)$, we systematically increased the time *t*_offset_ before starting to analyze each trajectory. With the exception of the shape parameter *γ* all the other parameters of the Hill function describing $\overset{-}{\text{RMSD}}\left(\Delta t\right)$ exhibit a distinctive dependence on the initial phase of the trajectories and thus indicate that biased estimates would result if the initial phase would be included in the analysis.

As pointed out in the introduction, the method proposed here combines the smoothing effect of averaging, while retaining the specificity of a precise time interval between configurations being compared. For example, the height of the extrapolated $\overset{-}{\text{RMSD}}$ plateau *a*_0_ (see Figure 6) may be interpreted as “configurational distance” between two arbitrarily selected, totally unrelated configurations of the particular part of phase space currently visited. If one chooses an arbitrary configuration out of it as the reference, all others visited by the trajectory will have an average distance *a*_0_. In contrast, Figure 1 shows two limiting cases of reference configurations: (i) the configuration with maximum average distance (i.e., RMSD) to all others (blue curve), which represents the “maximum outlier” ever seen in this trajectory and (ii) the configuration with minimum average distance (i.e., RMSD) to all others (red curve), which is “most central” within the trajectory.

Note that extrapolated plateau values are significantly larger than the $\overset{-}{\text{RMSD}}\left(\Delta t\right)$ actually reached in the trajectories (see Figure 3). Only if we consider the extrapolated plateau as a function of *t*_offset_, we obtain realistic (i.e., lower) estimates (see Figure 6) to aim at during actual simulations. In this sense, we may attribute the proposed fitting procedure some forecast capability regarding the level of RMSD to be finally expected if the trajectory were carried on. This extrapolated mean RMSD corresponds to pairs of configurations separated by time intervals large enough to consider such configurations approximately uncorrelated, independent representatives of the configuration space of the respective molecule.

Likewise, the “half-saturation time” *τ* obtained from the fit decreases with increasing *t*_offset_. Thus, taking configurations with large enough *t*_offset_ as a reference, it takes only a short time to get close to the $\overset{-}{\text{RMSD}}$ plateau *a*_0_. In each panel of Figure 5, the parameter *τ* approaches an almost constant level at about half the maximum *t*_offset_ considered (i.e., 25% of the total simulation time). This might suggest that—regardless of the total simulation length—the fraction of usable (independent) configurations remains the same (final 75% of the trajectory). As the length of the simulation run increases, the criterion derived from the run itself gets increasingly more stringent. From the 200 ns run the first 50 ns should be discarded, which is more than 5times the total length of the 10 ns trajectory. If using the latter as a basis of estimate, however, the last 7.5 ns seem to be trustable.

Although the analysis reported in the present work is specific to TCRpMHC complexes, we expect the method of lagged RMSD analysis to be applicable to similar molecular systems, such as membrane proteins comparable in size and structure. The approach presented here is designed to assess the degree of convergence of MD simulations and hence the statistical quality of conclusions drawn from such simulations.

## Figures and Tables

**Table 1:** Molecular dynamics simulation runs.

	*t*_max⁡_ (ns)	Δ*t*_config_ (ps)	Peptide	*n*
Trajectory1	10	3	FLRGRAYGL	3500
Trajectory2	50	50	FLRGRAYGL	1000
Trajectory3	200	50	FLRGRAYGL	4322
Trajectory4	10	3	FLRGRAAGL	3500
Trajectory5	50	50	FLRGRAAGL	1000
Trajectory6	200	50	FLRGRAAGL	4322
